# Temperature and the evolution of flower color: A review

**DOI:** 10.1002/ajb2.70106

**Published:** 2025-10-07

**Authors:** Elizabeth P. Lacey

**Affiliations:** ^1^ Department of Biology University of North Carolina Greensboro Greensboro 27402 USA NC

**Keywords:** abiotic, drought, evolution, flavonoid, flower color, fruit color, geographic variation, light, review, temperature

## Abstract

Flower colors brighten our natural world. How and why have they evolved? How might ongoing global warming alter their evolutionary trajectories? In this review, I examine the influence of ambient temperature on the evolution of flower color. Given the wide body of literature on pollinator‐mediated selection, I restricted the review to temperature‐mediated selection and interactions between temperature and two other abiotic factors, drought and light. I focus on flavonoid‐based colors because they are widespread, and their biosynthetic pathway is well characterized. Accumulated data suggest that temperature has been a selective factor in determining large‐ and small‐scale geographic patterns in species having genetically fixed flower color and in species with temperature‐sensitive plasticity in color. However, it is also clear that we have much to learn about direct and indirect selection on flower color related to ambient temperature and temperature's contributions to phylogenetic color patterns. Therefore, I conclude with questions to help advance understanding about temperature's role in past evolution and present and future changes arising from global warming.

Flower colors, which brighten the natural world and the foods that we eat, bring us great joy. How and why have they evolved? Historically, the dominant hypothesis has been that floral color has evolved in response to divergent selection pressures arising from different pollinators. Data from many studies are consistent with this hypothesis. Further, data suggest that pollinator behavior and color have coevolved and that over time, pollinators have selectively shaped the floral hues, intensities, and patterns that we see today (e.g., Stebbins, 1974; Faegri and van der Pijl, 1979; Weiss, 1995; Proctor et al., 1996; Fenster et al., 2004). Consequently, do we need to ask whether any other selective force might also be operating?

There are good reasons to ask this question. First, the coevolutionary hypothesis often cannot explain many types of floral color variations. For example, it cannot explain floral color diversification in wind‐pollinated species (e.g., conifers, Rudall, 2020) or differential fruit set (e.g., Yang et al., 2015). Second, there is limited evidence that it explains large‐ and small‐scale geographic variation in flower color (see below). Third, the hypothesis generally assumes that pollination is the limiting step in the reproductive process, which some studies indicate is not true (e.g., Jones et al., 1986; Lavi and Sapir, 2015). Fourth, pollinators do not always preferentially visit one color (e.g., Hannan, 1981). Also, pollinator behavior fails to explain how pleiotropic relationships between vegetative and floral traits may have influenced evolutionary changes in flower color (e.g., Armbruster, 2002). Consequently, biologists have suggested that factors other than pollinators have often contributed to floral color diversification (Armbruster, 2002; Strauss and Whittall, 2006; Rausher, 2008; Sobel and Streisfeld, 2013; Tripp et al., 2018, Vaidya et al., 2018).

One such factor is temperature. Many floral traits such as shape, size, color, UV protection, and thermogenesis are ecologically associated with thermal habitat (e.g., Kevan, 1989; Van der Kooi et al., 2019; Sapir et al., 2021). In this review, I focus on the evolutionary aspects of color and examine the hypothesis that prevailing temperatures during a species' reproductive season have often contributed to floral color diversification. Data relevant to this hypothesis are scattered in the literature. Some studies are descriptive; others are experimental. In some species, flower color is genetically fixed; in others, color is phenotypically plastic (i.e., genotypes modify their flower color in response to a changing ambient thermal environment during the flowering season). Here, I bring together this literature to evaluate the hypothesis that prevailing temperatures during a species' reproductive season have often influenced floral color diversification and to identify where more information is needed to improve our understanding of temperature's selective role. Space prevents an exhaustive literature review. Therefore, I mention representative studies and reviews that (1) illustrate important points and (2) provide entries to additional literature. My review focuses on thermal effects experienced by plants in populations along natural spatial and temporal gradients and is organized in sections addressing the following topics: spatial and temporal patterns in flower color, flavonoids, temperature, floral color and reproductive success, related abiotic factors: drought and light, pleiotropic interactions, and phylogenetic patterns. In my concluding remarks, I pose questions for future research.

## SPATIAL AND TEMPORAL PATTERNS IN FLOWER COLOR

Documented patterns of floral color variation associated with large‐scale geographic thermal gradients date back to the late 19th century. Bonnier (1888), Bonnier and Flahault (1878a), Bonnier and Flahault (1878b) and Flahault (1878) reported that the intensity of floral color in a species' population is often positively associated with latitude (Table 1). Pellat (1878) and Kerner von Marilaun and Oliver (1894) observed parallel associations with altitude (Table 2). Whereas whole flowers or inflorescences of some species (e.g., spikes, panicles) are darker at higher latitudes/altitudes, only a particular floral part appears darker (e.g., petals, bracts) in others. For some species, however, Kerner and Oliver noted that it is the vegetative color that intensifies at higher altitudes (Table 2). Such observations collected dust in libraries during the 20th century while evolutionary biologists focused on plant–pollinator coevolution. Only in the last few decades has interest in these geographic patterns renewed, with biologists identifying more species with similar patterns associated with thermal gradients and some with contrasting geographic patterns where color is negatively associated with latitude (Table 1).

**Table 1:** Documented latitudinal variation in flower color intensity and hue.

Family	Species	Latitudinal color change	Source
**A. Positive associations for flowers**			
Asteraceae	*Carduus crispus*	More brilliant pink or almost crimson	Bonnier (1888)
Asteraceae	*Cirsium arvense*.	More brilliant pink or almost crimson	Bonnier (1888)
Asteraceae	*Hieracium alpinum*	Darker yellow	Bonnier (1888)
Asteraceae	*Leontodon autumnalis*	Darker yellow	Bonnier (1888)
Campanulaceae	*Campanula persicifolia*	Darker blue	Bonnier (1888)
Campanulaceae	*Campanula rotundifolia*	Darker blue	Bonnier (1888)
Caprifoliaceae	*Scabiosa succisa*	Darker blue	Bonnier (1888)
Ericaceaee	*Calluna vulgaris*	More intense pink/almost crimson	Bonnier (1888)
Fabaceae	*Trifolium ratense*	More intense pink/almost crimson	Bonnier (1888)
Geraniaceae	*Erodium cicutarium*	More intense pink/almost crimson	Bonnier (1888)
Lamiaceae	*Origanum vulgare*	Darker crimson	Bonnier (1888)
Onagraceae	*Epilobium spicatum*	More intense violet	Bonnier (1888)
Papaveraceae	*Fumaria officinalis*	Darker crimson	Bonnier (1888)
Plantaginaceae	*Linaria vulgaris*	Darker yellow	Bonnier (1888)
Polygalaceae	*Polygala depressa*	More intense blue	Bonnier (1888)
Ranunculaceae	*Ranunculus glacialis*	Darker crimson	Bonnier (1888)
Saxifragales	*Saxifraga aixoides*	Orangish to red above 62°C	Bonnier (1888)
Violaceae	*Viola tricolor*	Intense violet	Bonnier (1888)
Plantaginaceae	*Plantago lanceolata*	Light green to brown/black	Lacey et al. (2010)
Campanulaceae	*Campanula americana*	More intense blue	Koski and Galloway (2020)
Onagraceae	*Clarkia unguiculata*	Petals: more intense purple	Peach et al. (2020)
**B. Positive associations for fruits**			
Ericaceaee	*Vaccinium vitis‐idaea*	More intense red	Bonnier (1888)
Rosaceae	*Cotoneaster vulgaris*	More intense red	Bonnier (1888)
Rosaceae	*Fragaria vesca*	More intense red	Bonnier (1888)
**C. Negative associations for flowers**			
Primulaceae	*Lysimachia arvensis*	Blue to orange	Arista et al. (2013)
Caryophyllaceae	*Silene littorea*	Dark pink to white	Del Valle et al. (2015)

**Table 2:** Documented altitudinal variation in flower color intensity and hue.

Family	Species	Plant part	Color change	Source
**A. Positive associations for flowers**				
Poaceae	*Aira caespitosa*	Spikes; panicles	Pale green to deep violet tint	Kerner von Marilaun and Oliver (1894)
Poaceae	*Briza media*	Spikes; panicles	Pale green to deep violet tint	Kerner von Marilaun and Oliver (1894)
Poaceae	*Festuca nigrescens*	Spikes; panicles	Pale green to deep violet tint	Kerner von Marilaun and Oliver (1894)
Poaceae	*Milium effusum*	Spikes; panicles	Pale green to deep violet tint	Kerner von Marilaun and Oliver (1894)
Poaceae	*Poa annua*	Spikes; panicles	Pale green to deep violet tint	Kerner von Marilaun and Oliver (1894)
Poaceae	*Poa nemoralis*	Spikes; panicles	Pale green to deep violet tint	Kerner von Marilaun and Oliver (1894)
Apiaceae	*Chaerophllum cicutaria*	Floral bracts	White to blue or red	Kerner von Marilaun and Oliver (1894)
Apiaceae	*Laserpitium latifolium*	Floral bracts	White to blue or red	Kerner von Marilaun and Oliver (1894)
Apiaceae	*Libanotis montana*	Flower	White to dark brownish violet	Kerner von Marilaun and Oliver (1894)
Apiaceae	*Libanotis montana*	Floral bracts	White to blue or red	Kerner von Marilaun and Oliver (1894)
Apiaceae	*Pimpinella magna*	Floral bracts	White to blue or red	Kerner von Marilaun and Oliver (1894)
Asteraceae	*Achillea millefolium*	Floral bracts	White to blue or red	Kerner von Marilaun and Oliver (1894)
Asteraceae	*Carlina acaulis*	Floral bracts	White to blue or red	Kerner von Marilaun and Oliver (1894)
Asteraceae	*Taraxacum officinale*	Flower	Lighter to darker	Kerner von Marilaun and Oliver (1894)
Brassicaceae	*Cardamine amara*	Floral bracts	White to blue or red	Kerner von Marilaun and Oliver (1894)
Campanulaceae	*Campanula pusilla*	Flower	Lighter to darker	Kerner von Marilaun and Oliver (1894)
Caryophyllaceae	*Agrostemma githago*	Flower	Lighter to darker	Kerner von Marilaun and Oliver (1894)
Caryophyllaceae	*Dianthus inodorus (sylvestris)*	Flower	Lighter to darker	Kerner von Marilaun and Oliver (1894)
Caryophyllaceae	*Gypsophylla repens*	Floral bracts	White to blue or red	Kerner von Marilaun and Oliver (1894)
Caryophyllaceae	*Gypsophylla repens*	Flower	Lighter to darker	Kerner von Marilaun and Oliver (1894)
Caryophyllaceae	*Saponaria ocymoides*	Flower	Lighter to darker	Kerner von Marilaun and Oliver (1894)
Cyperaceae	*Carex aterrima*	Floral bracts	Dark violet, almost black	Kerner von Marilaun and Oliver (1894)
Cyperaceae	*Carex atrata*	Floral bracts	Dark violet, almost black	Kerner von Marilaun and Oliver (1894)
Cyperaceae	*Carex nigra*	Floral bracts	Dark violet, almost black	Kerner von Marilaun and Oliver (1894)
Fabaceae	*Lotus corniculatus*	WF	Lighter to darker	Kerner von Marilaun and Oliver (1894)
Fabaceae	*Vicia cracca*	WF	Lighter to darker	Kerner von Marilaun and Oliver (1894)
Fabaceae	*Vicia sepium*	WF	Lighter to darker	Kerner von Marilaun and Oliver (1894)
Juncaceae	*Juncus castaneus*	Floral bracts	Dark violet, almost black	Kerner von Marilaun and Oliver (1894)
Juncaceae	*Juncus jacquinii*	Floral bracts	Dark violet, almost black	Kerner von Marilaun and Oliver (1894)
Juncaceae	*Juncus trifidus*	Floral bracts	Dark violet, almost black	Kerner von Marilaun and Oliver (1894)
Lamiaceae	*Satureja hortensis*	WF	Lighter to darker	Kerner von Marilaun and Oliver (1894)
Asteraceae	*Bellis perennis*	Flower	Very darker purple	Pellat (1878)
Asteraceae	*Hieracium sabinum*	Flower	Yellow lemon to orangish yellow	Pellat (1878)
Asteraceae	*Pilosella*	Flower	Yellow lemon to orangish yellow	Pellat (1878)
Boraginaceae	*Myosotis alpetris*	Flower	Yellow lemon to orangish yellow	Pellat (1878)
Boraginaceae	*Myosotis silvatica*	Flower	Yellow lemon to orangish yellow	Pellat (1878)
Campanulaceae	*Campanula linifolia*	Flower	Darker blue	Pellat (1878)
Campanulaceae	*Campanula rhomboidalis*	Flower	Darker blue	Pellat (1878)
Campanulaceae	*Campanula rotundifolia*	Flower	Darker blue	Pellat (1878)
Fabaceae	*Onobrychis montana*	Flower	Rose to crimson or purple	Pellat (1878)
Orchidaceae	*Orchis latifolia*	Flower	darker red/almost black violet	Pellat (1878)
Pinaceae	*Abies concolor*	Cone	Green to purple	Sturgeon and Mitton (1980)
Caryophyllaceae	*Silene vulgaris*	Calyx	Green to purple	Berardi et al. (2016)
Plantaginaceae	*Plantago lanceolata*	Bracts + flower	Light green to brownish black	Lacey et al. (2010)
**B. Positive associations for vegetative structures**				
Asteraceae	*Leucanthemum vulgare*	Leaves	Green to violet	Kerner von Marilaun and Oliver (1894)
Scrophulariaceae	*Pedicularis incarnata*	Leaflets; stems	Wholly purple or dark violet	Kerner von Marilaun and Oliver (1894)
Scrophulariaceae	*Pedicularis incarnata*	Leaflets; stems	Wholly purple or dark violet	Kerner von Marilaun and Oliver (1894)
Scrophulariaceae	*Pedicularis recutita*	Leaflets; stems	Wholly purple or dark violet	Kerner von Marilaun and Oliver (1894)
Scrophulariaceae	*Pedicularis rostrata*	Leaflets; stems	Wholly purple or dark violet	Kerner von Marilaun and Oliver (1894)
Crassulaceae	*Sedum altratum*	Leaflets; stems	Wholly purple or dark violet	Kerner von Marilaun and Oliver (1894)
Scrophulariaceae	*Bartsia alpina*	Leaflets; stems	Wholly purple or dark violet	Kerner von Marilaun and Oliver (1894)
Caryophyllaceae	*Lychnis viscaria*	Leaves	Green to brownish red	Kerner von Marilaun and Oliver (1894)
Crassulaceae	*Sedum acre*	Leaves	Green to purplish red	Kerner von Marilaun and Oliver (1894)
Crassulaceae	*Sedum album*	Leaves	Green to purplish red	Kerner von Marilaun and Oliver (1894)
Crassulaceae	*Sedum seangulare*	Leaves	Green to purplish red	Kerner von Marilaun and Oliver (1894)
Lamiaceae	*Dracocephalum ruschianum*	Leaves	Green to violet	Kerner von Marilaun and Oliver (1894)
Lamiaceae	*Satureja hortensis*	Leaves	Green to brownish red	Kerner von Marilaun and Oliver (1894)
Rosaceae	*Potentilla tiroliensis*	Leaves	Green to scarlet red	Kerner von Marilaun and Oliver (1894)
Saxifragaceae	*Bergenia crassifolia*	Leaves	Green to scarlet red	Kerner von Marilaun and Oliver (1894)
Papaveraceae	*Papaver radicatum*	Flower	White to yellow	Mølgaard (1989)

Abbreviation: WF = whole flower.

Floral color in some species changes spatially over small‐scale thermal gradients. In *Lotus corniculatus*, dark‐keeled plants tend to inhabit colder microsites (Jewell et al., 1994). In *Nigella degenii* subsp. *barbro*, which produces yellow or violet flowers, the frequency of the darker morph is higher on the north‐ and east‐facing slopes than on south‐ and west‐facing slopes (Jorgensen and Andersson, 2005). Dick et al. (2011) observed that the frequency of white‐flowered individuals in 14 populations of *Parrya nudicaulis* populations is positively correlated with temperature during the growing season in Alaska. Purple‐flowered individuals predominate in regions where the flowering season is colder.

**Figure 1:**
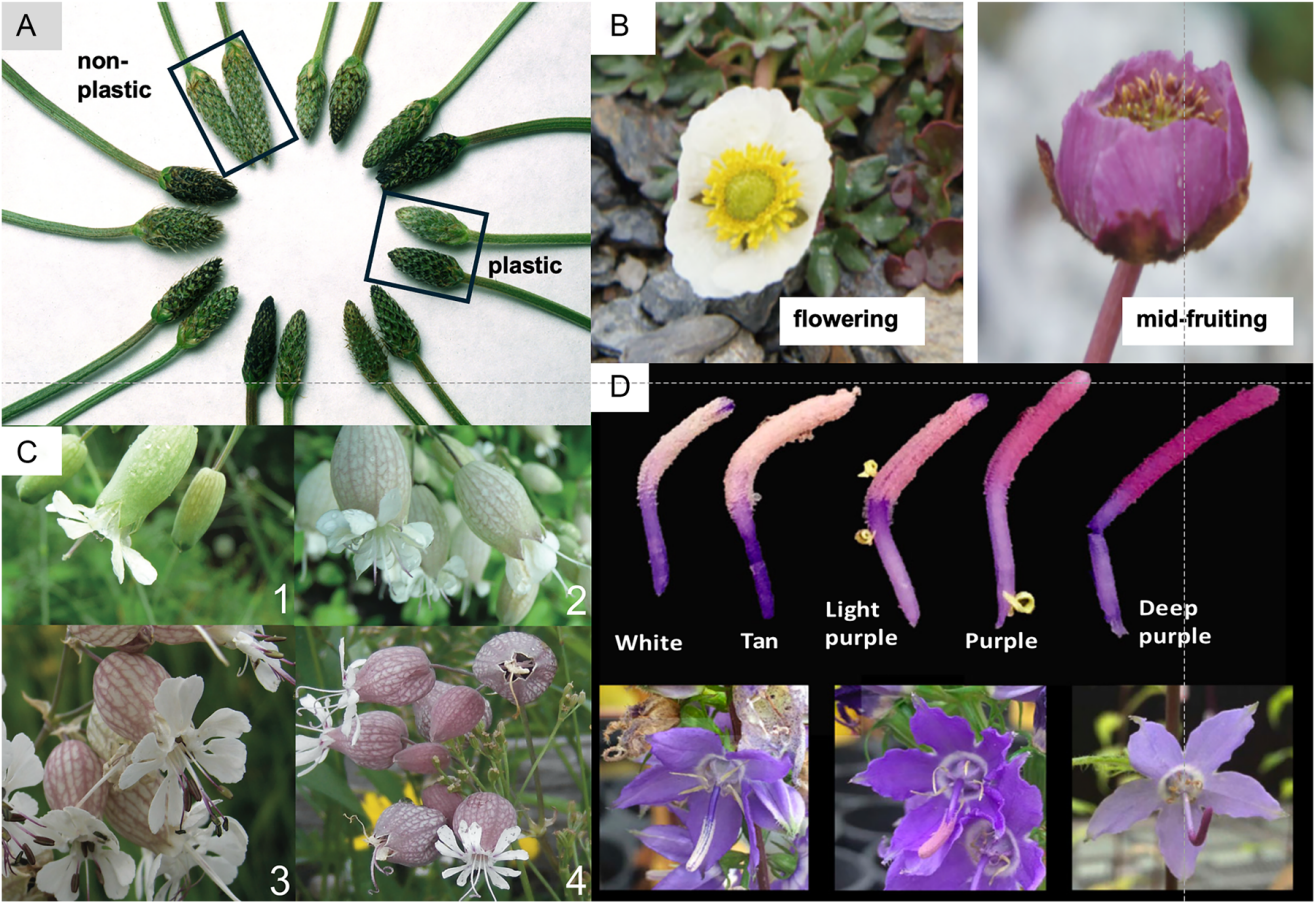
Examples of floral color differences in (A) inflorescences of thermally plastic and nonplastic *Plantago lanceolata* genotypes just before stigma emergence. From the perspective of the circle's center, the inflorescence on the right for each genotype was produced at ~7°C cooler temperature. (B) *Ranunculus glacialis* petals during flowering and mid‐fruiting stages (Ida and Totland, 2014), (C) anthocyanin calyx color classes (1 = no pigment to 4 = dark anthocyanin) in *Silene vulgaris* plants (Berardi et al., 2016), and (D) color of *Campanula americana* pollen deposited on styles removed from flowers and found on intact flowers (Koski and Galloway, 2018). All photographs are reprinted by permission of authors cited.

Also, botanists have observed that flower color within a population can change throughout a flowering season. Ambient temperatures typically change during the reproductive season (e.g., spring lows to summer highs and autumn lows in temperate regions). Negative associations between floral color and temperature have been reported for some species, whereas in other species, floral color and temperature are positively associated. Kerner von Marilaun and Oliver (1894) reported that the corolla in *Lamium album* (Lamiaceae) and ray petals of *Bellis perennis* (Asteraceae) darken with a seasonal drop in temperature. In *Ophiorrhiza japonica* populations, pink‐flowering plants are more abundant than white‐flowering plants in spring, but not in winter (Wang et al., 2024), and later, I offer Wang's explanation. In wind‐pollinated *Plantago lanceolata*, genotypes lighten the color of new spikes produced as monthly ambient temperature rises from spring to mid‐summer (Figure 1A; Lacey and Herr, 2005). The magnitude of this phenotypically plastic response is positively correlated with latitude and altitude (Lacey et al., 2010). In contrast, white flowers of *Ranunculus glacialis* (Figure 1B; Ida and Totland, 2014) and petals of *Gentiana leucomelaena* (Mu et al., 2010) flowers darken to purple or blue as the reproductive season progresses. Kerner von Marilaun and Oliver (1894) reported that several Asteraceae and Ranunculaceae species change color depending on whether ligulate florets or sepals, respectively, are open or closed during the flowering season (Table 3). Weiss (1995) and Weiss and Lamont (1997) found that, after a predictable number of days past floral opening in animal‐pollinated angiosperms from 78 families, individual floral color changes. Their assumption was that these developmental modifications evolved in response to pollinator behavior (e.g., Weiss, 1995), the rationale being that a plant differentiating between pollinated and unpollinated flowers increases its fitness because the efficiency of pollinator service increases. While that assumption may be true, authors did not consider alternatives causal hypotheses related to the abiotic environment. Flower color polymorphisms are increasingly being identified in species inhabiting the Mediterranean region (Narbona et al., 2018), but as is the case with most of the patterns identified above, the selective agents remain to be identified.

**Table 3:** Color differences between surfaces of floral structures.

Family	Species	Structure	Closed outer (abaxial) surface color	Open inner (adaxial) surface color	Source
Asteraceae	*Anacyclus officinarum*	Ligulate florets	Red, violet, or blue	White	Kerner von Marilaun and Oliver (1894)
Asteraceae	*Bellis perennis*	Ligulate florets	Red, violet, or blue	White	Kerner von Marilaun and Oliver (1894)
Asteraceae	*Calendula pluvialis*	Ligulate florets	Red, violet, or blue	White	Kerner von Marilaun and Oliver (1894)
Asteraceae	*Hieracium pilosella*	Ligulate florets	Yellow	White	Kerner von Marilaun and Oliver (1894)
Ranunculaceae	*Anemone alpina*	Sepals	Red, violet, or blue	White	Kerner von Marilaun and Oliver (1894)
Ranunculaceae	*Anemone baldensis*	Sepals	Red, violet, or blue	White	Kerner von Marilaun and Oliver (1894)
Ranunculaceae	*Anemone nemorosa*	Sepals	Red, violet, or blue	White	Kerner von Marilaun and Oliver (1894)
Ranunculaceae	*Anemone sylvestris*	Sepals	Red, violet, or blue	White	Kerner von Marilaun and Oliver (1894)
Ranunculaceae	*Anemone trifolia*	Sepals	Red, violet, or blue	White	Kerner von Marilaun and Oliver (1894)

In summary, the literature provides many documented examples of species in which floral color is negatively, or positively, associated with ambient temperature, e.g., at higher and lower latitudes and altitudes and at different times during a reproductive season (e.g., Tables 1, 2). Why do we see contrasting spatial patterns? Do these spatial and temporal patterns reflect genetically fixed differences among plants growing in different populations or flowering at different times within a population? Does variation in color pattern reflect phenotypic plasticity? What evolutionary mechanism(s) have produced these patterns? What are the pigments?

## FLAVONOIDS AND THE EFFECTS OF TEMPERATURE ON FLAVONOID PRODUCTION

**Figure 2:**
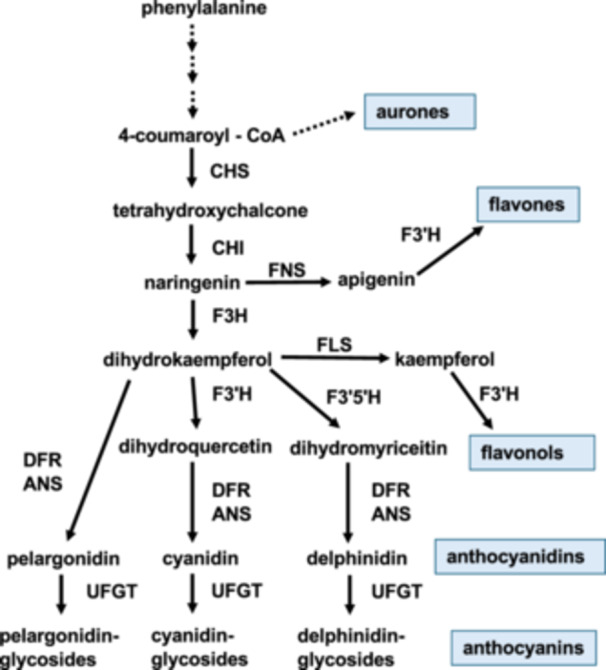
Generalized diagram of the flavonoid biosynthetic pathway. CHS = chalcone synthase; CHI = chalcone‐flavanone isomerase; FNS = flavone synthase; F3H = flavanone 3‐hydroxylase; F3′H = flavonoid 3′‐hydroxylase; F3′5′H = flavonoid 3′,5′‐hydroxylase; FLS = flavonol synthase; DFR = dihydroflavonol 4‐reductase; ANS = anthocyanidin synthase; UFGT = UDP flavonoid glucosyltransferase.

In trying to answer the above questions, it is useful to explore first what is known about flavonoid‐based flower color. Flavonoids strongly determine floral color in many plant species. The flavonoid biosynthetic pathway is well characterized (Figure 2), and recent advances in genomic technology have greatly improved our understanding about its genetic architecture. In this section, I briefly describe this pathway and what we know about temperature's influence on floral pigment production.

Anthocyanins, end products of the flavonoid pathway (e.g., Winkel‐Shirley, 2002; Grotewold, 2006; Davies et al., 2012), primarily determine the geographic variation in floral color in many angiosperms. For example, Narbona et al. (2018) recently surveyed flower color polymorphism in the Mediterranean Basin. They estimated that nearly 88% of floral color polymorphisms involved anthocyanins, although carotenoids may also contribute to floral color. Red, blue, and purple anthocyanin pigments are most immediately derived from one of three anthocyanidins: pelargonidin, cyanidin, and delphinidin, respectively. Flavonols, which are colorless or pale yellow, are the most important copigments (e.g., Iwashina, 2015; Luo et al., 2016). They, along with flavones, chalcones, and aurones, can interact with anthocyanins to modify floral color (Asen et al., 1972; Mol et al., 1998; Iwashina, 2015). For example, flavonols in lowland *Papaver radicatum* flowers explain their yellow color; higher‐altitude plants lacking flavonols produce white flowers (Wind et al., 1998). Lawrence (1932) found that ivory flavone can intensify the blue color of flowers in *Dahlia* and other genera. Also, pH, metal ions, tissue and epidermal cell structure, and sugar concentration can affect pigment color (e.g., Bao et al., 2020, Ma et al., 2024).

At least seven enzymatic reactions make up the structural backbone of the flavonoid pathway, and these are genetically determined by a well‐defined group of structural genes. The expression of these genes is controlled by regulatory genes. One group controls the production of transcription factors (TFs). Transcription factors regulate structural genes through a MYB‐bHLH‐WDR complex of proteins, and the large R2R3‐MYB family disproportionally determines floral anthocyanin variation (e.g., Quattrocchio et al., 2006; Albert et al., 2014). Transcription factors can work as activators, repressors, or both to regulate the expression of target genes (Chen et al., 2022). For example, a single MYB TF represses anthocyanin production in white flowering *Iochroma loxense* (Gates et al., 2018). Individual copies of R2R3‐MYBs are often tissue‐specific, such that a single copy may regulate pigmentation specifically in flowers, while another copy regulates pigmentation in the vegetative tissues.

Anthocyanins and copigments contribute to flower color in two ways. They determine hue and intensity. The anthocyanin biosynthetic pathway (ABP) branches, e.g., cyanidin branch controlled by the enzyme flavonoid 3' ‐hydroxylase (Figure 2), functionally determine color, or hue (e.g., red vs. blue vs. purple). Studies of agronomically and horticulturally important species and species growing in natural populations shows that flower hue is often controlled by one or a few genes (e.g., in *Brassica* species, review by Ma et al., 2021). Extensive work in model organisms such as *Petunia*, *Antirrhinum*, *Mimulus*, and *Ipomoea* has demonstrated that loss‐of‐function (LOF) mutations in structural and regulatory genes typically change floral hue, e.g., from blue to red, or pigmented to white (e.g., Holton and Cornish, 1995; Mol et al., 1998, Streisfeld et al., 2013). Loss of pigment production has historically been associated with inactivation of one or more anthocyanin branches or through recruitment of a LOF mutation or a flavonoid pathway inhibitor (e.g., Grotewold, 2006; Rausher, 2008). A LOF mutation of a structural gene can result in a new genetically fixed color because the mutation inactivates a pathway/branch, which may be difficult to reactivate (Rausher, 2008). Additionally, gain‐of‐function mutations in a repressor are now known to also inhibit pigment production (Gates et al., 2018). Currently, there is little evidence that temperature underlies variation in flower hue in natural populations. However, experimental evidence from *Antirrhinum majus* suggests that temperature can induce mutations for a flower‐hue gene and that these new alleles can be temperature sensitive (Harrison and Fincham, 1964).

In contrast to hue, the amount of flux through an ABP branch (i.e., branch activity) determines intensity (Rausher, 2008; Sobel and Streisfeld, 2013). Many controlled experiments of agronomically and horticulturally important species (e.g., *Malus*, *Vitis*, *Prunus*) show that ambient temperature during flowering and fruiting strongly influences intensity by modifying the expression of structural and regulatory genes in inbred or artificially selected lines. Typically, high‐temperature stress causes the fading of flower and fruit color, whereas cool‐temperature stress increases color intensity (reviews: Jaakola and Hohtola, 2010; Gu et al., 2019; Ahmad et al., 2022; also see Zoratti et al., 2015; Naing et al., 2018; Alcantud et al., 2023; Wu et al., 2024). Increased pigment intensity can occur when anthocyanins are already present, either by removing the repression of a TF that inhibits expression, upregulating a TF that promotes expression, or by increasing structural gene expression through changes in *cis*‐regulatory regions (review by Yan et al., 2021). In other words, there are multiple ways by which an environmental factor could modify pigment production. Shvarts et al. (1997) found that low temperature induces corolla pigmentation and *CHS* expression in *Petunia* in the presence and absence of light. Other data from *Petunia* show that such a simple change in flower hue and intensity is not always as simple (e.g., does not always involve a LOF mutation; Berardi et al., 2021).

Knowledge about temperature control of pigment production in natural plant populations is very limited. Cooler temperatures increase anthocyanin production, mostly cyanidin derivatives, in *P. lanceolata* (Stiles et al., 2007). Heritable temperature‐sensitivity (i.e., the ability to darken flowers) is positively correlated with latitude and altitude (Lacey and Herr, 2005; Lacey et al., 2010; Marshall et al., 2020). Lu et al. (2009) found that temperature also differentially affected cyanidin‐based anthocyanins levels in *Ipomoea purpurea* plants from natural populations in China. *Silene vulgaris* produces more floral anthocyanins in cooler, high‐altitude populations than in warmer, low‐altitude populations (Figure 1C; Berardi et al., 2016). Notably, the reverse pattern was detected in leaves, where non‐anthocyanin flavonoids were positively associated with altitude. Luo et al. (2016) proposed that relative differences in FLS and DFR expression, which lead to flavonols and anthocyanins, respectively, determine whether seven commercially important species produce white or red flowers. Whether such a model applies to nature populations remains to be tested.

In summary, 19th century botanists reporting spatial and temporal variation in flower color had no knowledge of the genetic basis for these patterns, and this is still true for the species that they observed, with one exception (*Lotus corniculatus*, Ramnani and Jones, 1984). The flavonoid pathway was unknown. Today, we know that for many species, color variation is genetically based. Also, the flavonoid pathway is well characterized. However, knowledge about their environmental and genetic controls is still quite limited.

## TEMPERATURE, FLOWER COLOR, AND REPRODUCTIVE SUCCESS

Spatial and temporal patterns identified in Part A suggest that in multiple species, population variation in flower color reflects past adaptive evolution in response to variation in local thermal environment. Support for this hypothesis comes from ecological studies showing that flower color influences the internal flower temperature, thereby improving a plant's reproductive success (reviewed by Kevan et al., 2019 and Van der Kooi et al., 2019). Here, I expand on their information. I focus on studies that concern temperatures during reproduction, rather than annual mean temperature values for a species, because I believe that temperature during the reproductive season is more likely to influence flower color evolution in a species.

First, studies show that ambient temperature may influence different types of pollination and affect one or more reproductive stages. It can influence both pollinator attraction (e.g., visits: Orueta, 2002; Figueroa‐Castro and Cano‐Santana, 2004; composition: Antonini et al., 2005) and wind pollination (e.g., Rodríguez‐Rajo et al., 2005; Kuparinen et al., 2009). Temperature can affect pollen and ovule development, fertilization, seed development, and offspring quality via transgenerational effects (e.g., Roach and Wulff, 1987; Cerović and Ružić, 1992; Egea and Burgos, 1995; McKee and Richards, 1998a, b; Cerović et al., 2000; Lacey and Herr, 2000; Hedhly et al., 2003; Borghi et al., 2019). In *Prunus avium*, temperature affects stigmatic receptivity (Hedhly et al., 2003). With experimental warming, stigmas first lose the capacity to sustain pollen tube penetration, then to support pollen germination, and finally to retain pollen on the surface. In *Tilia cordata*, a small change of 2–3°C can sharply alter pollen germination from near 0 to 70% (Pigott and Huntley, 1981). Pollen tube growth can affect siring ability, competitive ability, and even self‐compatibility (e.g., Dane and Melton, 1973; Gawel and Robacker, 1986). Experimental demonstrations of temperature effects are diverse in method and show that affected species are phylogenetically and ecologically diverse (e.g., review by Hedhly, 2011).

Second, reproductive stages can differ in thermal performance curves and thermal optima. In general, thermal performance curves vary within and between species, as for pollen‐tube growth in *Betula pendula* (Pasonen et al., 2000). Unpredictable and wide temperature swings during a flowering season may select for wide thermal performance curves, as in the high‐altitude *Gentianella germanica* (Steinacher and Wagner, 2012). Stage‐specific differences in optima and range and in the thermal environment during the flowering period can help explain why color polymorphisms in many species are isolated to a particular floral structure (e.g., petals: Levin and Brack, 1995; Schemske and Bierzychudek, 2001; Irwin and Strauss, 2005; perianth and stigmas: Rafiński, 1979; pollen: Jorgensen and Andersson, 2005; Koski and Galloway, 2018) (Figure 1D). Also, stage‐specific differences may produce conflicting fitness effects. Koski et al. (2020) made controlled crosses of *Campanula americana* and observed that dark pollen grains were larger than lightly colored grains and that plants producing dark pollen sired more seeds with greater germination potential. However, plants with lightly colored pollen produced more seeds per fruit. They suggested that the color of different floral structures, e.g., gynoecium vs. androecium, may produce antagonistic effects.

Given the potential ways by which flower color can mediate the impact of ambient temperature on reproductive success, it is easy to understand why researchers have proposed that ambient temperatures experienced by plants during their reproductive seasons have contributed to spatial and temporal patterns of floral color in natural populations. Underlying this hypothesis, however, is the assumption that floral color does, in fact, modify the internal floral thermal microenvironment in a manner that improves reproductive success, i.e., overall fitness.

Results of multiple studies are consistent with this assumption. The arctic heliotropic species *Papaver radicatum* produces either white or yellow flowers, and yellow flowers are 1.4–1.7°C warmer than white flowers on sunny days (Mølgaard, 1989). This thermal effect of color on internal floral temperature likely explains why yellow‐flowered plants predominate in sunnier but colder higher‐elevation populations, whereas white‐flowered plants are found in cloudier but warmer lowland coastal populations in Greenland. In arctic and alpine species, elevating internal flower temperature can offset low ambient temperatures and hasten reproductive development to maximize reproductive output. In flowers of *Lotus corniculatus*, the keel encloses the reproductive organs and can be yellow or brown (Jewell et al., 1994). On cool sunny days, internal temperatures of brown‐keeled flowers are consistently warmer, which likely helps to explain why dark morphs are more frequent in populations in cooler microhabitats (e.g., north‐facing slopes, higher altitudes). Sturgeon and Mitton (1980) observed that the frequency of purple to green cones increases with increasing altitude in *Abies concolor* and that purple cones get warmer than do green cones. In *Crocus*, purple‐ and white‐flowered species are warmer than yellow‐flowered species at the same ambient temperature, and they also support greater pollen germination and pollen tube growth (McKee and Richards, 1998b).

Predictable temperature change during a reproductive season appears to selectively favor temperature‐sensitive plasticity in floral color in some species (i.e., favor genotypes that modify floral color as a reproductive season progresses). Petal color in the alpine species *Ranunculus glacialis* changes from white to purple as a flower closes and transitions from pollination to fruit development (Figure 1B). Ida and Totland (2014) found that purple petals, which enclose developing achenes, raise the thermal microenvironment of developing achenes more than do white petals, which may improve seed production in an alpine environment. In *Gentiana leucomelaena*, another alpine species, some genotypes produce white flowers early in the flowering season when ambient temperatures are cooler and purple flowers later in warmer conditions (Mu et al., 2010). Anthers in white flowers get warmer than in purple flowers in the same ambient conditions. In the wind‐pollinated *P. lanceolata*, internal flower temperatures warm more in darkly colored spikes than in lightly colored spikes in the same sunny conditions, and this warming improves seed production at cool temperature (Lacey and Herr, 2005; Lacey et al., 2012).

Field and controlled experiments provide evidence that heritable temperature‐sensitive plasticity in color is favored in some years in mid‐latitudes populations of *P. lanceolata* (Lacey and Herr, 2005; Lacey et al., 2003, 2012; Marshall et al., 2020). When grasshopper populations were low, Lacey et al. (2012) transplanted clones of genotypes varying in temperature‐sensitivity (highly sensitive [plastic] to insensitive [nonplastic]) into the field. The clones had been induced in growth chambers to phenotypically express floral color at both warm and cool temperatures before transplantation during the normal flowering season. Plastic and nonplastic genotypes began flowering at the same time (mid‐April), but April seed production per spike was higher for plastic genotypes in normally cool temperatures. July production was higher than in April and similar for light‐colored spikes from both plastic and nonplastic genotypes in normally warm temperatures. Summed over the whole reproductive season, fecundity between plastic and nonplastic genotypes did not differ. However, in some years, large grasshopper populations feed heavily on maturing spikes in summer, greatly reducing summer seed production (Lacey et al., 2003), thereby providing plastic genotypes, which produce more seeds early, a fitness advantage. Temperature and herbivory together appear to maintain populations of *P. lanceolata* that are genetically variable for level of temperature‐sensitive plasticity in floral color at mid‐latitudes.

Finally, population divergence in floral color along thermal environmental gradients is generally assumed to have arisen because of adaptation. However, neutral processes, e.g., genetic drift, limited gene flow, could alternatively explain divergence. Two studies have tested these alternatives. One concerned latitudinal variation in degree of temperature‐sensitivity in spike color of *P. lanceolata* (Marshall et al., 2019). The other addressed the association between altitude and calyx color in *Silene vulgaris* flowers (Berardi et al., 2016). For both species, phenotypic differentiation–neutral genetic differentiation comparisons provide stronger support for natural selection than for neutral selective agents.

In summary, spatial and temporal patterns of flower color in many species are consistent with the hypothesis that darker floral pigments improve reproductive success against cool/cold stress, although experimental tests in the field are rare. In contrast, dark floral color in a few species is positively correlated with warmer latitudes (Table 1). Does floral pigmentation provide reproductive advantages at both ends of a typical thermal performance curve, albeit for different reasons? Do other abiotic factors function as selective agents for flower color? Is floral color a byproduct of direct selection acting on vegetative color?

## RELATED ABIOTIC FACTORS: DROUGHT AND LIGHT

Abiotic factors such as incoming solar UV‐B radiation, general light levels, precipitation, salinity, daylength, and temperature vary across the Earth's surface (e.g., Nybakken et al., 2004; Jaakola and Hohtola, 2010), and more than one may selectively act on flower color in natural plant populations. At a global scale, different abiotic stressors appear to select for distinct flower colors (see Dellinger et al., 2025, in this issue). Most information about these factors, other than temperature, concerns drought (i.e., precipitation) and light.

Drought, in addition to temperature, has been proposed as a selective agent that influences the geographic distributions of floral color patterns in multiple species. In *Lysimachia arvensis*, the blue‐flowered morph has higher fitness than the red morph in xeric environments and is more common in drier Mediterranean areas, whereas the red morph performs better in wet environments and is more abundant in wetter zones (Arista et al., 2013). In the Alps, calyx color intensity of *Silene vulgaris* negatively covaries more with altitudinal variation in precipitation and temperature than with UV radiation, although the correlation with temperature is stronger (Berardi et al., 2016). For 12 North American polymorphic species, color intensity is positively associated with aridity and negatively associated with temperature (Sullivan and Koski, 2021). *Erythranthe discolor* and *Diplacus mephiticus* (Phrymaceae) are both polymorphic for floral color. Grossenbacher et al. (2021) found that pink flowers are more abundant in arid *Erythranthe* populations, whereas in *Diplacus*, pink flowers are more abundant in regions of greater aridity and lower temperatures and UV radiation. Synergistic effects of drought and temperature have been suggested in a genome‐wide study of *Nicotiana tabacum* (Yang et al., 2023). Researchers found that *NtUGT* genes associated with differently colored flowers, are differentially expressed when plants are subjected to drought and low temperature.

Other studies focusing solely on drought provide additional support that precipitation is an important selective factor in nature. Schemske and Bierzychudek (2001) observed that blue‐flowered morphs of *Linanthus parryae* are more fit than white‐flowered morphs during years of drought, but that white‐flowered morphs are more fit during years of high spring precipitation. Pollinators showed no preference for a morph, and the morphs showed no difference in water‐use efficiency. Warren and Mackenzie (2001) observed that pigmented morphs of five British species consistently produce more seeds than do white‐flowered morphs in drought conditions, whereas the reverse is true in well‐watered conditions. Field and greenhouse experiments with *N. degenii* subsp. *barbro* showed that dark‐pollen genotypes have higher mortality under drought stress and nutrient deficiency than do light‐pollen genotypes (Andersson and Jorgensen, 2005; Jorgensen and Andersson, 2005). This difference may help to explain why dark morphs frequent north‐ or east‐facing slopes rather than south‐ or west‐facing slopes. Experimentally induced drought increases purple floral color in self‐pollinating *Boechera stricta*, which could explain the greater frequency of purple‐flowered genotypes at lower elevations (Vaidya et al., 2018). Drought, herbivory (greater for white flowers), and soil minerals all appear to influence the altitudinal distribution of floral color in this species.

The effects of light on flower pigmentation have been demonstrated in many studies (e.g., Dong et al., 1998; Mol et al., 1998; Farzad et al., 2003; Albert et al., 2009). Blocking natural light before flower bud break substantially lowers anthocyanin levels, resulting in pure white, rather than dark pink flowers in apple (Dong et al., 1998). Del Valle et al. (2015) observed that *Silene littorea* calyx color intensifies toward the south on Spain's west coast, paralleling increases in UV‐B radiation, aridity, and temperature. Color intensity and anthocyanin concentration in floral calyxes are positively correlated with temperature and solar exposure but negatively correlated with rainfall. Also, a controlled sun/shade experiment showed that anthocyanins increase when plants are exposed to more sun. (Del Valle et al., 2018). In *Clarkia unguiculata*, floral anthocyanin concentration is highest in populations experiencing high solar UV radiation (Peach et al., 2020). However, Albert et al. (2009) determined that anthocyanin levels in flowering heads of *Arnica montana* is better explained by colder temperatures than by enhanced UV‐B at higher altitudes. Lu et al. (2009) tested the effects of temperature, moisture, and UV intensity on anthocyanin content in *Ipomoea purpurea* in greenhouse and field experiments and found that temperature most strongly influences anthocyanin content.

Many conclusions drawn about the effects of light on pigmentation and reproductive success should be viewed cautiously. The reason is that “light” has typically been framed in terms of what our eyes can detect (excluding studies of UV patterns and pollinator behavior). This visible (VIS) region represents only a small portion (300–700 nm) of the electromagnetic spectrum. Thermal effects of light depend more strongly on absorbance of near‐infrared (NIR) wavelengths in the 780–2500 nm range, which our eyes cannot detect (Stuart‐Fox et al., 2017). In terms of solar radiation, about 55% of the radiant energy in direct sunlight falls within the NIR range, while around 45% falls within the VIS range. On a sunny day, most incoming solar radiation hitting the Earth's surface lies in the NIR and IR regions (Gates, 1980). On cloudy days, clouds capture so much NIR and IR solar radiation that the proportional representation of VIS radiation hitting the Earth's surface is greatly increased.

Few studies of flower color have measured reflectance across the electromagnetic spectrum. The earliest that I have found is a study by McKee and Richards (1998a) of four *Crocus* varieties that differ in petal color. Reflectance data from the UV to the NIR shows that *Crocus* has the typical attributes of “micro‐greenhouse” flowers. Inner tepal surfaces are highly reflective and more so in the NIR than are outer surfaces. Thus, the inner surfaces reflect most NIR wavelengths inward toward the central developing reproductive cells, likely warming them. Outer surfaces absorb more incoming light and, therefore, can warm flowers when closed. Lacey and Herr (2005) measured reflectance in the UV to NIR regions in *P. lanceolata* spikes and observed that cool temperature reduces reflectance (i.e., increases absorbance) in both VIS and NIR regions, but not in the UV. This temperature sensitivity provides partial thermoregulatory ability by warming reproductive cells during spring and cooling cells during summer. Koski and Galloway (2020) also measured reflectance in *Campanula americana* flowers across the UV‐NIR spectrum. While they did not find evidence for thermoregulatory ability, they did find that flowers from cooler, northern latitude populations absorb more in the VIS and NIR region than do those from southern populations (Figure 1D).

## PLEIOTROPIC INTERACTIONS

In 2002, Armbruster proposed that floral color diversification can evolve because of pleiotropic relationships between floral color and vegetative traits. In other words, direct selection on a vegetative trait can produce indirect selection on a floral trait, and one not necessarily favored by selection (see also Frey, 2004; Strauss and Whittall, 2006; Rausher, 2008; Sobel and Streisfeld, 2013; Narbona et al., 2018). Pleiotropy can stem from a single gene (e.g., structural, TF) that activates multiple downstream branches of the flavonoid pathway (see Figure 2). Thus, a mutation in a single gene could produce a cascade of phenotypic effects by modifying the activity of multiple flavonoid branches in multiple plant tissues.

Flavonoids probably arose in vegetative tissues long ago in response to abiotic stressors, and these tissues often retain stress‐related functions (Winkel‐Shirley, 2002; Koes et al., 2005; Buer et al., 2010). For example, leaves can reduce cellular and molecular heat stress by increasing leaf reflectance and pigment level. Both help dissipate high thermal energy, thereby improving water uptake and decreasing transpiration (e.g., Tattini et al., 2000; Tafesse et al., 2022, Satyakam et al., 2023). Correlated responses may also occur in flowers. Flavonoids (including anthocyanins, flavones, flavonols) provide protection from drought (e.g., Dabravolski and Isayenkov, 2023; Tenhumberg et al., 2023) and damaging effects of UV‐B radiation (Nybakken et al., 2004; Treutter, 2006) by counteracting reactive oxygen species (ROS) damage—possibly also in flowers. ROS trigger the activity of MYB transcription factors associated with the flavonoid pathway (e.g., Gould, 2004; Lai et al., 2011; Wu et al., 2024), and anthocyanins can function as photoreceptors, antioxidants, and osmoregulators, helping plants survive under multiple forms of intense abiotic stress (e.g., Steyn et al., 2002, Gould, 2004, Landi et al., 2015).

The degree of coupling of floral and vegetative pigments has been controversial. Armbruster's (2002) phylogenetic analyses suggest that coupling has driven evolutionary divergence between species of *Dalechampia* (Euphorbiaceae) and *Acer* (Aceraceae). Coupling may also have occurred in *Mimulus lewisii*. Pink‐flowered *M. lewisii* produces cyanidin in its leaves and flowers, but pigments are absent from both leaf and floral tissues of white‐flowered plants (Wu et al., 2013). Many British species polymorphic for floral color produce more deeply pigmented flowers and stems when subjected to water stress (Warren and Mackenzie, 2001). In the study by Berardi et al. (2016), anthocyanins in flowers and flavones in leaves strongly covaried with temperature and precipitation along an elevational gradient. Coupling of floral pigments with flowering time may be common. Timing of a flowering season is commonly triggered by reliable environmental cues, such as day length and temperature (Rathcke and Lacey, 1985). Species naturally cued to flower at stressful temperatures (e.g., in spring or autumn, at high altitudes, under heat stress) appear to produce more anthocyanins than do species cued to flower when ambient temperatures are less stressful (McKee and Richards, 1998a; Arista et al., 2013; Ilk et al., 2015; Keerthi et al., 2016; Brutch et al., 2019).

In contrast, a survey by Wessinger and Rausher (2012) indicates that floral and vegetative pigment production are more often decoupled than coupled. They found that floral anthocyanidin production is highly variable (20% pelargonidin, 44% cyanidin, 45% delphinidin), whereas nearly 90% of the same species produce only dehydroxylated cyanidins in vegetative tissue. In yellow‐flowered *Diplacus* (*Mimulus*) *aurantiacus*, anthocyanidins are produced in stems but not flowers (Streisfeld and Rausher, 2009). *CHS* expression in white‐flowered individuals of *Parrya nudicaulis* is blocked in petals, but not in leaves (Dick et al., 2011). *Silene littorea* increases anthocyanins in calyces and stems, but not petals or leaves in sunny conditions (del Valle et al., 2018). When shifted from warm‐ to cool‐temperature growth chambers, newly produced spikes of *P. lanceolata* reduce reflectance in VIS and NIR regions, but newly produced rosette leaves do not (Lacey et al., 2012). Different MYB copies typically activate anthocyanins in separate tissues (Stracke et al., 2001; Morita et al., 2006; Quattrocchio et al., 2006; Schwinn et al., 2006; Gonzalez et al., 2008), which would help explain why decoupling is common.

Coupling may be a mixed bag (Berardi et al., 2016). The adaptive advantage of pleiotropy, i.e., coupling vs. decoupling, depends on the relative costs and benefits of each phenotypic response and their contributions to individual fitness (Guillaume and Otto, 2012). Because the flavonoid pathway has many steps, even one environmental factor, such as temperature, may produce multiple responses, Thus, the fitness consequences of coupling seem likely to be quite diverse.

## PHYLOGENETIC RELATIONSHIPS

Environmental induction of genes underlying evolutionary transitions among species is largely unexplored. Rausher's survey (2008) suggested that color transitions are equally likely to be driven by abiotic and biotic factors. I have found only two studies examining temperature effects using a phylogenetic perspective. Anderson et al. (2013) measured temperature‐sensitive floral plasticity in nine species spanning a wide range of the *Plantago* phylogeny. All showed evidence of temperature‐sensitive floral plasticity in both VIS and NIR regions, suggesting that temperature‐sensitivity is an ancestral trait for this genus. However, the patterns of plasticity differed between species. Sánchez‐Cabrera et al. (2024) compared blue–orange floral color polymorphisms between *Lysimachia monelli* and *L. arvensis*. Based on nearly identical flavonoid biochemistry, gene expression patterns, and reflectance spectra in the UV and VIS regions, polymorphism likely reflects an ancestral trait for these species.

## CONCLUDING REMARKS

Central to the evolution of flower color is the importance of abiotic factors in determining color and in driving evolutionary change. Ecological studies have documented how color can contribute to a flower's thermal microenvironment, thereby influencing reproductive success. Advances in genomic technology have provided valuable information about the genetic mechanisms by which color in crop and non‐crop species is determined by the flavonoid pathway and its alterations. This information provides a strong foundation for addressing the many temperature‐relevant evolutionary questions that persist. Here I identify questions, whose answers, I believe, can help advance our understanding of temperature's role in floral color evolution. Many apply equally well to other abiotic factors.

(1) Many species show spatial and temporal variation in flower color associated with thermal gradients at large and small geographic scales, but only a few species have been studied to clarify the causes of this variation. To what extent do these patterns reflect population‐level variation in genetically fixed colors, temperature‐sensitivity in color, or both? Does floral pigmentation of a population improve fitness in its local environment? To what extent do temperature and biotic factors (e.g., pollinators, herbivores) interact antagonistically and/or synergistically?
(2) Information about temperature regulation of the flavonoid pathway is limited, though data suggest that *cis*‐ and *trans*‐regulatory gene expression is involved. Currently, data indicate that temperature affects floral intensity rather than hue. What mechanisms underlie this regulation? What mechanisms constrain responses to temperature? How do vegetative and reproductive structures mechanistically differ in temperature sensitivity?
(3) Data suggest that color and an internal floral temperature may differentially affect pollen, ovule, and seed development. Also, temperature may differentially affect reproductive vs. vegetative structures. Are these fitness effects synergistic or antagonistic? How does selection on color in one organ affect the adaptation of color in another?
(4) Solar radiation includes a range of electromagnetic wavelengths, which can induce different responses in plants. In contrast to the visible region of the electromagnetic spectrum, the near‐infrared region more strongly determines heat absorption. How do spatial and temporal patterns in visible and near‐infrared regions covary within and across species? How cloudy is a reproductive season? How do flowers modify the incoming radiation reaching reproductive cells?
(5) Most experiments testing the effect of temperature on color and reproductive success have been done in controlled conditions such as greenhouses and growth chambers. How does ambient temperature experienced by a population during flowering affect color and fitness of the population in its natural habitat?
(6) Geographic variation in natural environments is typically characterized by variation in temperature, precipitation, light, and other factors. What are the relative impacts of each factor on color? What are their pleiotropic interactions? Do the pleiotropic effects change geographically?
(7) Little is known about the influence of temperature on floral color at the macroevolutionary level. Do general and replicated patterns of temperature‐related genetic control appear in large taxonomic groups? To what extent are these patterns predictable and potentially useful for predicting responses to global climate change?

Answers to the above questions are important not only for understanding temperature's role in floral color evolution, but also for understanding how global climate change is likely to alter floral color. Current global trends are likely to alter the reproductive success in crop and non‐crop plant species because ambient temperature affects the thermal environment of all steps in the reproductive process of producing fruits and seeds. Finding answers to these questions will help us predict plant responses to climate change and counteract deleterious effects of ongoing changes.
